# Effects of Glucagon-Like Peptide-1 on Oxidative Stress and Nrf2 Signaling

**DOI:** 10.3390/ijms19010026

**Published:** 2017-12-22

**Authors:** Yoon Sin Oh, Hee-Sook Jun

**Affiliations:** 1 Department of Food and Nutrition, Eulji University, Seongnam 13135, Korea; ysoh@eulji.ac.kr; 2 College of Pharmacy and Gachon Institute of Pharmaceutical Science, Gachon University, Incheon 21936, Korea; 3 Lee Gil Ya Cancer and Diabetes Institute, Gachon University, Incheon 21999, Korea; 4 Gachon Medical Research Institute, Gil Hospital, Incheon 21565, Korea

**Keywords:** oxidative stress, reactive oxygen species, Nrf2 signaling, diabetes, glucagon-like peptide-1

## Abstract

Oxidative cellular damage caused by free radicals is known to contribute to the pathogenesis of various diseases such as cancer, diabetes, and neurodegenerative diseases, as well as to aging. The transcription factor nuclear factor erythroid 2-related factor 2 (Nrf2) and Kelch-like ECH-associated protein1 (Keap1) signaling pathways play an important role in preventing stresses including oxidative and inflammatory stresses. Nrf2 is a master regulator of cellular stress responses, induces the expression of antioxidant and detoxification enzymes, and protects against oxidative stress-induced cell damage. Glucagon-like peptide-1 (GLP-1) is an incretin hormone, which was originally found to increase insulin synthesis and secretion. It is now widely accepted that GLP-1 has multiple functions beyond glucose control in various tissues and organs including brain, kidney, and heart. GLP-1 and GLP-1 receptor agonists are known to be effective in many chronic diseases, including diabetes, via antioxidative mechanisms. In this review, we summarize the current knowledge regarding the role of GLP-1 in the protection against oxidative damage and the activation of the Nrf2 signaling pathway.

## 1. Introduction

Reactive oxygen species (ROS) and free radicals contribute to oxidative stress in healthy cells by damaging DNA, RNA, proteins, and lipids. Oxidative stress results from an imbalance between pro-oxidants and antioxidants, when the endogenous antioxidant system cannot properly remove free radical production. The damage caused by free radicals leads to a variety of diseases such as diabetes, diabetic complications, cancer, and neuronal disorders [1,2,3,4]. Antioxidants play an important role in cellular defense against ROS and free radicals and, subsequently, in the prevention of disease. Antioxidants can scavenge ROS or increase the ability to neutralize ROS by inducing the expression of genes involved in cytoprotection [5]. Nuclear factor erythroid 2-related factor 2 (Nrf2) transcription factor is a key regulator in the redox balance and signaling and regulates the expression of many antioxidant and detoxification genes by binding to antioxidant response elements (AREs) [6,7,8,9,10,11,12,13]. As Nrf2 signaling is considered to be an important contributor to various disease conditions associated with oxidative damage, therapies targeting the Nrf2 signaling pathway represent a promising avenue in current research.

Glucagon-like peptide-1 (GLP-1) is an incretin hormone that is produced mainly by the enteroendocrine L cells in the distal intestine in response to nutrient ingestion [5,14,15]. Upon binding to its receptor, GLP-1 affects blood glucose levels by stimulating insulin secretion, inhibiting glucagon secretion, inhibiting gastric emptying, and reducing food intake [16,17,18]. Because of its actions on the control of blood glucose, GLP-1 is now widely used in the clinic for diabetic patients [19,20]. In addition to its hypoglycemic effect, GLP-1 has anti-inflammatory, antioxidative, neurogenerative, and vascular protective effects in various cells and tissues including the kidney, lung, heart, hypothalamus, endothelial cells, neurons, astrocytes, and microglia, as well as pancreatic beta cells [21,22,23,24]. GLP-1 and GLP-1 receptor (GLP-1R) agonists also influence various cellular pathways including inhibition of inflammation and apoptosis, and protection against oxidative stress.

In this review, we will summarize the current knowledge regarding the role of GLP-1 in the protective effects against oxidative stress and the Nrf2 signaling pathway.

## 2. Oxidative Stress and Nuclear Factor Erythroid 2-Related Factor 2 (Nrf2) Signaling

ROS are generated in peroxisomes and mitochondria by normal cellular metabolism or are induced by exogenous stimuli such as inflammatory cytokines, chemical oxidants, ionizing radiation, and toxins. Under homeostasis, ROS are maintained at low levels by balancing their production and scavenging contributing to normal physiological function [25]. However, the failure of scavenging mechanisms or insufficient antioxidants results in incomplete oxidation and subsequent production of excess ROS, such as superoxide anion (O_2_^−^), hydroxyl radical (-OH), and hydrogen peroxide (H_2_O_2_). Excess ROS damage nucleotides, proteins, and lipids and make these biomolecules nonfunctional [26,27,28,29,30]. Although ROS are important signaling molecules for various biological effects, excessive ROS cause oxidative stress and contribute to aging and many pathological conditions, including cancer, diabetes, neurological disorders, atherosclerosis, hypertension, ischemia/perfusion, and asthma [31,32,33,34,35,36,37,38,39,40,41]. Antioxidant systems include enzymatic and nonenzymatic antioxidants that are usually effective in neutralizing highly reactive ROS. Nonenzymatic antioxidants include glutathione, ascorbic acid, and tocopherol and many phenolic compounds from natural products [42].

**Figure 1:**
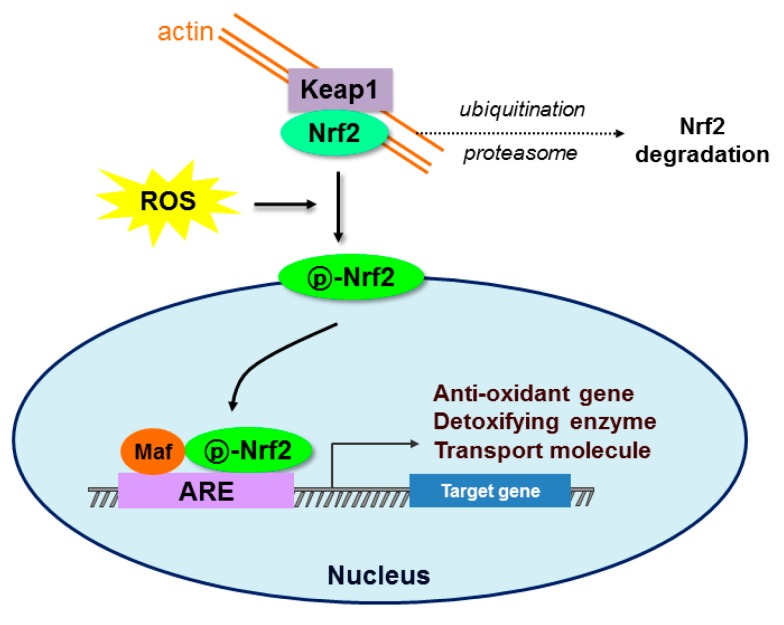
Schematic diagram of the Nrf2-Keap1-ARE signaling pathway. Under normal conditions, nuclear erythroid-2 like factor-2 (Nrf2) is constantly ubiquitinated through Kelch-like ECH-associated protein1 (Keap1) and degraded in the proteasome. After exposure to oxidative stress (ROS), Keap1 is inactivated and Nrf2 becomes phosphorylated. Phosphorylated Nrf2 (p-Nrf2) accumulates in the nucleus and binds to antioxidant response element (ARE) sites, subsequently activating many genes including antioxidants, detoxifying enzymes, and transport molecules.

A key molecule regulating the cellular antioxidant response is Nrf2, a member of the cap ”n” collar subfamily of basic region leucine zipper transcription factors. Under basic conditions, Nrf2 is bound to the endogenous inhibitor Kelch-like ECH-associated protein1 (Keap1), which is anchored to actin in the cytoskeleton in the cytosol, and suppressed by Keap1-dependent ubiquitination-proteasomal degradation [8]. The Keap1 protein contains several cysteine residues with sulfhydryl groups that can react with ROS, causing the bonds between Nrf2 and Keap1 to break. Once the bonds are broken, Nrf2 becomes phosphorylated at Ser 40 and translocates to the nucleus of the cell [43]. In the nucleus, it forms heterodimers with other transcription factors such as c-Jun and small Maf proteins and binds to regulatory regions of DNA, called an ARE, that turn on the transcription of genes coding for antioxidant enzymes. c-Jun acts mainly as a transcriptional activator, while the small Mafs inactivate gene transcription after Nrf2 binding [44]. Nrf2–ARE binding regulates the expression of genes involved in cellular antioxidant and anti-inflammatory responses such as superoxide dismutase, catalase, glutathione peroxidases, thioredoxin, thioredoxin reductase, sulfiredoxin, NADPH:quinone oxidoreductase-1, heme oxygenase-1, glutathione reductase, glutaredoxin, glutamate cysteine ligase, glutathione S-transferase, UDP-glucuronyl transferase, peroxiredoxin sulfotransferase, and γ-glutamate cysteine ligase catalytic subunit [45] (Figure 1).

In addition to their location in the cytoskeleton, Nrf2 and Keap1 have also been detected at the outer mitochondrial membrane, binding to mitochondrial phosphatase phosphoglycerate mutase (PGAM) family member 5 [46] and affecting mitochondrial function. Nrf2 activates the expression of genes promoting mitochondrial biogenesis and preservation, such as mitochondrial transcription factors, mitochondrial DNA-directed RNA polymerase, citrate synthase, NADH dehydrogenases, and Cox subunit 1 [47,48]. Moreover, Nrf2 promotes p62 (an autophagic adaptor protein sequestosome-1) expression, which is involved in mitophagy (maintains the mitochondrial integrity by removing damaged mitochondria) and helps maintain mitochondrial homeostasis [48,49].

In addition, the expression of over 200 genes including phase I and II detoxification enzymes, transport proteins, proteasome subunits, chaperones, growth factors and their receptors, as well as some other transcription factors, are regulated by the Nrf2/ARE pathway [45]. Knockout of Nrf2 in mice increased their susceptibility to various chemical toxicity and disease conditions associated with oxidative pathologies such as type 2 diabetes, cancer, cardiovascular disease, and neurodegenerative disease and also aging [8,50]. Moreover, pharmacological activation of Nrf2 by various chemoprotective agents protects against oxidative damage [51].

Nrf2–ARE signaling is affected by crosstalk with several other signaling systems. Protein kinases and phosphatases also regulate Nrf2 signaling, as phosphorylation of serine residues in Nrf2 enables it to enter the nucleus. These protein kinases and phosphatases include mitogen-activated protein kinase (MAPK), casein kinase 2, the protein kinase R-like endoplasmic reticulum kinase, protein kinase (PK) C, the sarcoma (Src) family of protein kinases, glycogen synthase kinase-3, adenosine monophosphate-activated kinase, phosphatidylinositide 3-kinase (PI3K), and AKT [52].

## 3. Glucagon-Like Peptide-1 (GLP-1)

GLP-1 is an incretin hormone, which is secreted from the intestine in response to food ingestion. The main effect of GLP-1 is to stimulate insulin secretion from pancreatic islets in a glucose-dependent manner. GLP-1 has many additional effects; it delays gastric emptying, inhibits food intake, improves insulin sensitivity, inhibits glucagon secretion, and stimulates insulin biosynthesis [18,53].

### 3.1. Synthesis and Metabolism

The proglucagon gene is expressed in enteroendocrine L cells in the small and large intestines, in the central nervous system, and in pancreatic alpha cells [54]; different peptides are produced by cell-specific differential post-translational processing of the proglucagon protein [55]. Processing of proglucagon by prohormone convertase 2 in pancreatic alpha cells produces glucagon (the major product), glicentin-related polypeptide, intervening peptide-1, and major proglucagon fragment. Processing of proglucagon by prohormone convertase 1/3 in the gut and brain produces GLP-1, GLP-2, oxyntomodulin, glicentin, and intervening peptide-2 [55], and GLP-1 is the major peptide generated from proglucagon in the intestine.

GLP-1 in the circulation is rapidly increased by nutrients such as carbohydrates, fats, proteins, and dietary fiber and is truncated and amidated into two active forms: GLP-1 (7–37) and GLP-1 (7–36) amide [14]. Circulating GLP-1 is rapidly degraded by dipeptidyl peptidase (DPP)-4 and produces GLP-1 (9–37) and GLP-1 (9–36) amide, which is largely inactive but has been recently shown to regulate cardiovascular function [56]. These peptides can be further cleaved to generate GLP-1 (28–36) amide and GLP-1 (32–36) amide. Emerging evidence indicates that GLP-1 metabolites also have many beneficial effects, such as inhibition of hepatic glucose production, cardio- and neuroprotective effects, reduction of oxidative stress in the vasculature, and both antiapoptotic and proliferative effects in pancreatic beta cells [57].

GLP-1 binds to the GLP-1R, a seven-transmembrane guanine nucleotide-binding protein-coupled receptor, and activates multiple signaling pathways including cyclic adenosine monophosphate (cAMP)–protein kinase A (PKA), MAPK, epidermal growth factor receptor (EGFR)–PI3K, and PKB to exert various biological effects. GLP-1R is widely expressed in various tissues including pancreatic islets, pancreatic ducts, kidney, lung, heart, skin, immune cells, and the central and peripheral nervous systems, hypothalamus, hippocampus, and the cortex [58].

### 3.2. Pancreatic Effects

The primary physiological action of GLP-1 is to stimulate insulin secretion in pancreatic beta cells in a glucose-dependent manner. GLP-1-induced insulin secretion is mainly regulated by cAMP–PKA and exchange proteins activated by cAMP (Epac)2 signaling pathways [59]. GLP-1 also increases proinsulin gene expression, biosynthesis, and mRNA stability. The increase of proinsulin gene transcription by GLP-1 is mediated by upregulation of the expression of pancreas duodenum homeobox 1, a beta cell transcription factor [60]. cAMP/PKA-dependent and -independent signaling pathways and Ca^2+^ signaling pathways are also involved in insulin gene transcription by GLP-1 [61,62,63]. In addition, GLP-1 inhibits glucagon secretion in pancreatic alpha cells through a somatostatin-dependent mechanism [64].

Studies show that GLP-1 increases beta cell proliferation and neogenesis and inhibits beta cell apoptosis, contributing to the regulation of the beta cell mass [65,66]. The increase in the beta cell mass in 70% pancreatectomized mice was significantly lower in GLP-1R-deficient mice compared with wild-type mice, indicating an important role of GLP-1 in beta cell regeneration [67]. PI3K, PKC, PKB, c-Src, and MAPK signaling pathways mediate GLP-1-induced pancreatic beta cell proliferation [68,69]. Transcription factor 7-like 2 and Wnt/β-catenin signaling pathways are also involved in GLP-1-induced insulin secretion and beta cell proliferation [70,71].

Many studies have reported on the preventive effects of GLP-1 and GLP-1R agonists on beta cells against various toxic stimuli including glucose, fatty acids, cytokines, and ROS [65]. The anti-apoptotic effects of GLP-1 are mediated by increasing the anti-apoptotic proteins Bcl-2 and Bcl-xl and decreasing active caspase-3 [72] through cAMP and activation of PI3K, PKB, and EGFR–PI3K signaling pathways [73,74,75].

GLP-1 or exendin-4 treatment can convert rat or human pancreatic exocrine cells into glucagon-, insulin-, or pancreatic polypeptide-containing cells, and acinar pancreatic cells can differentiate into insulin-producing cells through PKC, MAPK signaling, and pancreatic and duodenal homeobox 1 (Pdx-1) regulation [76]. In addition, exendin-4 induces pancreatic duct cells to differentiate into insulin- or glucagon-secreting endocrine cells through induction of hepatocyte nuclear factor 3β and Pdx-1 [77].

### 3.3. Extrapancreatic Effects

In addition to its actions on the pancreas, GLP-1 reduces glucose production in the liver and increases glucose uptake in adipose tissue and muscle [64,78,79]. Activation of GLP-1R signaling enhances neurogenesis, reduces apoptosis, and protects neuronal function [80,81], suggesting that GLP-1 could be beneficial for the treatment of neurodegenerative disorders [82]. GLP-1R activation affects renal function [83] and has pleiotropic cardioprotective effects [84]. GLP-1-based therapy also shows anti-inflammatory effects not only on pancreatic islets but also on many other tissues such as adipose tissue, liver, brain, endothelial cells, kidney, and skin by reducing inflammatory cytokine production and infiltration of immune cells [24,85,86,87,88,89]. Thus, GLP-1 might have therapeutic potential for the treatment of nonalcoholic steatohepatitis, neurodegenerative disorders, atherosclerosis, diabetic nephropathy, and psoriasis. However, the clinical significance of GLP-1 in these conditions has not been determined yet.

## 4. Antioxidant Effect of GLP-1 in Diabetes

Increased oxidative stress plays a major role in the development and progression of diabetes and its complications [90]. Increased production of free radicals and ROS and impaired antioxidant defense accompany both type 1 and type 2 diabetes [91]. Pancreatic islets are particularly vulnerable to oxidative damage due to low expression levels of antioxidant enzymes [92].

**Figure 2:**
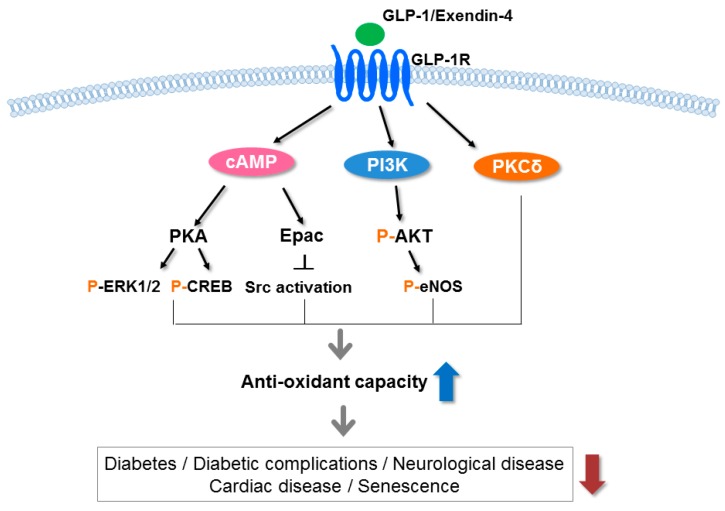
Signaling pathways underlying the antioxidative effects of the GLP-1 receptor. GLP-1 and exendin-4 (a GLP-1 receptor agonist) bind to the GLP-1 receptor (GLP-1R) and stimulate cyclic adenosine monophosphate (cAMP), phosphoinositide 3-kinase (PI3K) and protein kinase C (PKC)δ, subsequently activating a number of pathways including protein kinase A (PKA), exchange protein kinase activated by cAMP2 (Epac2) and protein kinase B (AKT). These pathways increase the antioxidant capacity in various tissues and reduce diabetes, diabetic complications, neurological disease, cardiac disease, and senescence. p, phosphorylation; ERK, extracellular signal-regulated kinase; CREB, cAMP response element binding protein; Src, sarcoma; eNOS, endothelial nitric oxide synthase 3.

GLP-1 has an antioxidative action as treatment of GLP-1 or its receptor agonist shows significant reductions in oxidative stress markers (superoxide dismutase, glutathione reductase, catalase, glutathione peroxidase, glutathione levels, lipid peroxidation, and nonenzymatic glycosylated proteins) induced by various stress factors. The mechanism by which GLP-1 decreases oxidative stress in diabetes was found to be via receptor-mediated activation of cAMP, PI3K, and PKC pathways and activation of Nrf-2, which consequently increases the antioxidant capacity (Figure 2).

Nrf2 knockdown in a mouse insulinoma cell line (MIN6) or pancreatic islets isolated from Nrf2-knockout mice are vulnerable to arsenic-induced cell damage [93], and pharmacological activation of Nrf2 improves islet yield and islet function after transplantation [94], indicating that Nrf2 plays a significant role in the protection of pancreatic beta cells from oxidative stress. In addition, Nrf2 activation improves insulin sensitivity in diabetes, abrogates diabetes and obesity in mice, and increases oxygen consumption and glucose uptake in skeletal muscle [95,96]. These results suggest that Nrf2 activation via GLP-1 and subsequent antioxidative action may be beneficial for the prevention and treatment of diabetes.

### 4.1. In Vitro Studies

Many in vitro studies confirm the protective effects of GLP-1 against oxidative stress. Glycated serum increased intracellular ROS production, reduced the expression of Nrf2, and decreased insulin content in a hamster pancreatic beta cell line (HIT-T15). However, co-treatment with GLP-1 (10 nmol/L) reversed these effects, suggesting that GLP-1 protects cells against oxidants [97]. *Tert*-butyl hydroperoxide-induced oxidative stress was reduced by GLP-1 treatment (10 nmol/L) in INS-1 pancreatic beta cells. Enhanced cellular levels of glutathione and the activity of its related enzymes—glutathione-peroxidase and -reductase—was observed in these GLP-1-treated cells, and the cAMP–mediated PKA/extracellular signal-regulated kinase (ERK) pathway was involved in antioxidant enzyme regulation [98]. Incubation of INS-1 cells with cytokines increased apoptosis via high ROS generation, and this was completely prevented after preincubation with the GLP-1R agonist, exendin-4 [99]. Glucose-induced ROS production and Src phosphorylation in islets of diabetic Goto–Kakizaki rats were significantly decreased by exposure to exendin-4 (100 nmol/L). When treated with an Epac-specific cAMP analog (9CPT-2Me-cAMP), Src phosphorylation and ROS production were decreased, suggesting that Epac-mediated Src inactivation is involved in the antioxidant effect of exendin-4, but not PKA or MAPK/ERK signaling pathways [100]. Kim et al. reported that exendin-4 (10 nmol/L) treatment decreased palmitate- or H_2_O_2_-induced ROS production and restored cellular glutathione levels and insulin secretion in INS-1 cells. The author demonstrated that PKCδ-mediated Nrf2 activation contributed to the increase in antioxidant gene expression and consequently improved beta cell function in the presence of oxidative stress [101] (Figure 2). GLP-1 (28-36) amide (100 nmol/L) also inhibited ROS formation and restored cellular ATP levels, therefore suppressing glucose production in hepatocytes isolated from high fat diet-induced diabetic mice [102]. These in vitro data suggest that GLP-1 exerts antioxidant effects by reducing ROS and increasing the antioxidant capacity via Nrf2 activation and induction of antioxidant enzymes.

### 4.2. In Vivo Studies

The beneficial effect of GLP-1 therapy in type 1 and type 2 diabetic animal models is due, in part, to its antioxidant activity. Recombinant human GLP-1 (24 nmol/kg/d) reduced hyperglycemia in streptozotocin (STZ)-induced diabetic mice, and superoxide dismutase and glutathione peroxidase activities were enhanced in the pancreas [103]. Administration of exenatide (1 μg/kg/d) for 10 weeks to STZ-induced diabetic rats resulted in reduced blood glucose levels and enhanced insulin release from the pancreas. Moreover, an increased number of catalase- and glutathione reductase-positive cells in pancreas was observed [104]. Shimoda et al. reported that the GLP-1 analog, liraglutide (400 μg/kg/d, 2 weeks), preserved pancreatic beta cells in diabetic db/db mice, and the expression of antioxidative stress genes, including catalase and glutathione peroxidase, was significantly increased in pancreatic beta cells [105].

Treatment with exendin-4 (10 or 20 μg/kg/d, 60 days) reversed hepatic steatosis in *ob*/*ob* mice by improving insulin sensitivity, and the level of thiobarbituric reactive substances as a marker of oxidative stress was significantly reduced in liver tissue of these mice [106]. A combination of omeprazole (30 mg/kg/d) and exendin-4 (8 μg/kg/d) treatment for four weeks in STZ-induced diabetic mice improved blood glucose levels compared with either exendin-4 or omeprazole alone, which was correlated with reduced liver lipid peroxidation and increased Nrf-2 expression [107]. As hyperglycemia or obesity may affect the oxidative stress status, it is difficult to determine the independent effect of blood glucose-lowering or body weight loss.

### 4.3. Clinical Studies

Clinical studies in diabetic patients show that short- and long-term treatment with GLP-1 reduces hyperglycemia and oxidative stress. Administration of GLP-1 (0.4 pmol/kg/min) for 2 h reduced 8-iso-prostaglandin F2α (8-iso-PGF2α) and nitrotyrosine (oxidative stress markers) in plasma during a hypoglycemia or hyperglycemia clamp in both type 1 and type 2 diabetic patients [108,109]. In a two-month prospective pilot study, administration of liraglutide (1.2 mg/d) to type 2 diabetic patients reduced serum lipid hydroperoxides and heme oxygenase 1, as well as significantly reduced glycated hemoglobin [110]. One year of treatment with exenatide (10 μg/d) reduced postprandial glycemia and lipidemia in type 2 diabetic patients, and these effects were related to decreased malondialdehyde (a lipid peroxidation marker) and oxidized low-density lipoproteins [111]. However, different results were observed in another study: eight months of liraglutide treatment (0.74 mg/d) did not affect serum levels of malondialdehyde [112]. This discrepancy might be due to the lower dose of liraglutide used.

## 5. Antioxidant Effect of GLP-1 in Diabetic Complications

A large number of in vitro and some in vivo studies have shown that the antioxidative effects of GLP-1 are involved in protecting against diabetic complications. With respect to effects on cardiac tissue, low GLP-1 levels and high nitrotyrosine (a ROS-dependent oxidative stress marker) levels were associated with cardiac remodeling and development of cardiovascular disease in type 2 diabetic patients [113]. In HL-1 cardiomyocytes, GLP-1 (100 and 200 nmol/L) reduced palmitate-induced cytosolic and mitochondrial oxidative stress and increased ATP synthase expression. Moreover, GLP-1 restored mitochondrial membrane permeability and cytochrome c oxidase activity, consequently inhibiting oxidative damage in mitochondria [113]. Treatment with exendin-4 (20 nmol/L) in neonatal rat cardiomyocytes attenuated H_2_O_2_-induced ROS production and increased the synthesis of antioxidant enzymes such as catalase, glutathione peroxidase-1, and manganese superoxide dismutase, and the effect was dependent on GLP-1R-mediated Epac pathways [114]. Increased apoptosis, mitochondrial dysfunction, and ROS production in cardiomyocytes (H9C2) by H_2_O_2_ and hypoxia/reoxygenation were ameliorated by exenatide (1 and 200 nmol/L), and the effect was associated with activation of the PI3K/AKT signaling pathway [115,116].

Endothelial damage via high glucose and/or ROS production by multiple biochemical pathways is an important contributor to cardiovascular disease. High glucose induces ROS production, increases the apoptotic index, and increases the level of NADPH oxidases such as p47^phox^ and gp91^phox^ in cardiac microvascular endothelial cells. These effects were inhibited after treatment with GLP-1 (10 nmol/L); an increased cAMP/PKA and decreased Rho expression were also demonstrated in this study [117]. GLP-1 (0.3 nmol/L) blocked the upregulation of vascular cell adhesion molecule (VCAM)-1 mRNA levels by advanced glycation end products in human umbilical vein endothelial cells. This effect was mediated through the GLP1R-cAMP axis, resulting in a lower expression level of the receptors for advanced glycation end products and ROS generation [118]. Exposure of human aortic endothelial cells to high glucose activated the PKC-NADPH oxidase pathway, evidenced by increased intracellular diacylglycerol and p47^phox^ translocation. A combination of liraglutide (30 nmol/L) and metformin inhibited these pathways and reduced oxidative stress [119].

High fat diet-induced diabetic cardiomyopathy, as evidenced by myocardial fibrosis and steatosis, was reduced by treatment with exendin-4 (24 nmol/kg/d, 40 days). Moreover, exendin-4 ameliorated myocardial oxidative stress via suppression of the ROS generating enzyme, nicotinamide adenine dinucleotide phosphate (NADPH) oxidase 4, with concomitant elevation of antioxidant enzymes (superoxide dismutase-1 and glutathione peroxidase) [105]. STZ- and high fat diet-induced diabetic rats showed a significant increase in oxidative damage of the aorta detected by NADPH oxidase 4 and VCAM-1 expression, and these increases were reversed by exenatide (10 μg/kg) treatment [120].

Diabetic nephropathy-prone KK/Ta-Akita mice exhibit increased albuminuria and mesangial expansion, as well as upregulation of glomerular superoxide and renal NADPH oxidase. However, treatment with liraglutide (200 μg/kg/d) for four weeks suppressed the progression of nephropathy, and this effect was mediated by an elevation in cAMP and PKA levels [22]. Hendarto et al. also reported that treatment of STZ-induced diabetic rats with liraglutide (0.6 mg/kg/d) for four weeks reduced oxidative stress markers (urinary 8-hydroxy-2′deoxyguanosine and renal dihydroethidium staining) in renal tissue and consequently reduced diabetic nephropathy [121]. The author demonstrated that PKA-mediated inhibition of renal NADPH oxidase was involved in protecting against diabetic nephropathy in STZ-induced diabetic rats [121]. Treatment with sitagliptin (a DPP-4 inhibitor, 10 mg/d for 20 weeks) decreased the expression of antioxidant response genes (peroxiredoxin and glutathione S-transferase) in the kidney from diabetic Goto-Kakizaki rats, and downregulation of miR-200a was involved in the protective effect [122].

## 6. Antioxidant Effect of GLP-1 in Neurological Diseases

As GLP-1 agonists can cross the blood–brain barrier, the effect of GLP-1 treatment on cellular pathways involved in neuroinflammation, mitochondrial function, neuronal protection, and cellular proliferation within the central nervous system have been investigated [123]. Cerebral ischemia-reperfusion injury in mice caused increased expression of oxidative stress markers (8-hydroxy-deoxyguanosine and inducible nitric oxide), apoptotic DNA fragmentation and infarct volume, and higher neurological deficit scores. After exendin-4 (10 μg/kg) treatment, the intracellular cAMP level was increased and neuroprotective effects were observed in these mice [124]. In a rat model of cerebral ischemia, long-lasting (at least 2 weeks) exendin-4-loaded microspheres exerted neuroprotective functions via reduced oxidative injury and endoplasmic reticulum(ER) stress. Nuclear factor (NF)-κB p65 and p-AKT/endothelial nitric oxide synthase (p-AKT/p-eNOS) pathways were involved in the neuroprotective effect [125]. Rats pretreated with liraglutide (50 μg/kg) for 14 days had a smaller infarct volume and decreased neurological deficit after middle cerebral artery occlusion-induced cerebral ischemia compared with ischemic controls. The pretreatment blocked the elevated levels of lipid peroxidation markers and decreased the activity of antioxidant molecules such as glutathione and superoxide dismutase seen in brain tissue of ischemic controls [126]. Zhu et al. demonstrated that liraglutide (500 nmol/L) inhibited oxygen/glucose deprivation apoptosis by reducing ROS in primary neurons and increasing the phosphorylation of AKT and ERK while decreasing phosphorylation of p-38 and c-Jun N-terminal kinase [127]. Moreover, liraglutide (100 μg/kg/d) reduced the infarct volume and improved motor and somatosensory function in ischemic rats, suggesting that liraglutide exerts neuroprotective actions against ischemia-induced apoptosis via activation of the PI3K/AKT and MAPK pathways [127]. Liraglutide (50 nmol/L) treatment increased cell proliferation in human-derived neuroblastoma cells via cAMP-response element binding protein phosphorylation. Moreover, pretreatment with liraglutide rescued cells from H_2_O_2_- or glutamate-induced cell death and led to behavioral improvements in a mouse model of traumatic brain injury [128]. These results indicate that GLP-1-based therapies could be a therapeutic treatment option for neurological diseases that are associated with oxidative stress.

## 7. Antioxidant Effect of GLP-1 in Senescence

Many theories on aging have been proposed, and the free-radical theory suggests that cumulative damage to mitochondria and mitochondrial DNA caused by ROS is one of the important causes of aging and aging-related diseases [33]. Several studies have shown that GLP-1 has protective effects against cellular senescence. GLP-1 (10 nmol/L) treatment inhibited H_2_O_2_-induced DNA damage and cellular senescence in human umbilical vein endothelial cells, and DPP-4 inhibition using vildagliptin (3 mg/kg/d) protected against vascular senescence in Zucker diabetic fatty rats [129]. The author suggested that the GLP-1R-mediated PKA pathway and induction of antioxidant genes such as heme oxygenase-1 and quinone oxidoreductase-1 were involved in this effect. Zhao et al. also demonstrated that the cAMP/PKA-dependent pathway was involved in the vascular anti-aging effect of GLP-1. Treatment with angiotensin II increased senescence-associated beta-galactosidase staining as well as levels of p53 and p21 in vascular smooth muscle cells of rat aorta, and the induction was mediated by superoxide anion generation from NADPH oxidase. Pretreatment with exendin-4 (10 nmol/L) blocked the angiotensin-induced premature senescence and H_2_O_2_ generation by inhibiting Ras-related C3 botulinum toxin substrate 1 (Rac1) activation via the cAMP/PKA-dependent pathway [130].

## 8. Conclusions

**Figure 3:**
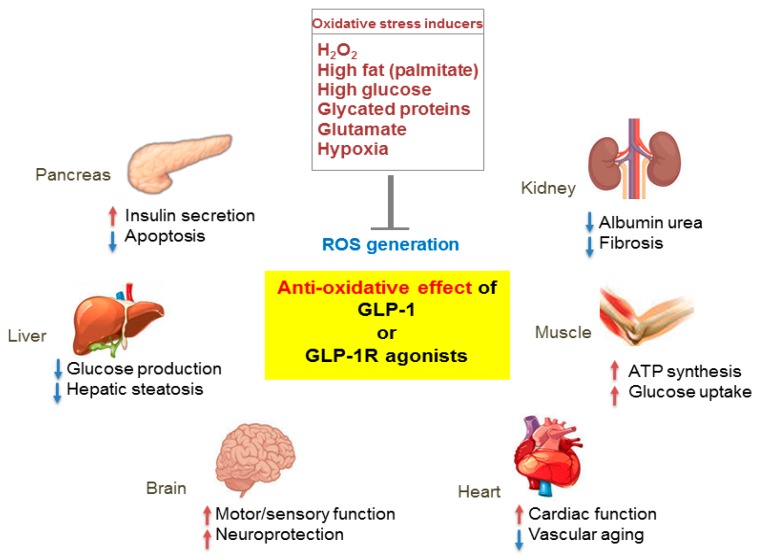
Pleiotropic effects of GLP-1 based therapies on antioxidant defense mechanisms in several organs. GLP-1 receptor (GLP-1R)-mediated signaling blocks reactive oxygen species (ROS) generation induced by various oxidative stressors and regulates the physiological function of various organs including the pancreas, liver, brain, heart, muscle, and kidney. Red arrows, increase; blue arrows, decrease.

Cumulative evidence indicates that ROS and oxidative stress promote the pathogenic process of various chronic diseases including diabetes, diabetes complications, neurological disorders, and cancer. The Nrf2–ARE pathway is a key system for antioxidative effects, and thus might be a logical therapeutic target for the prevention and treatment of disease. In addition to its glucose-lowering effect, in vitro and in vivo studies have proven that GLP-1 and GLP-1R agonists reduce ROS and protect against oxidative stress induced by various stress factors, such as high glucose, fatty acids, cytokines, and hydrogen peroxide, by enhancing the expression of antioxidant enzymes and activating Nrf2 (Figure 3). Although the reduction of ROS and activation of Nrf2 pathways are glucose-independent effects of GLP-1, the antioxidant effects of GLP-1 in diabetes or diabetes complications might be attributed in part to its glucose lowering effect, which reduces oxidative stress. Most of the results obtained are from in vitro and animal studies, and very limited data are available from clinical studies. Further studies are needed to understand the detailed mechanisms for the antioxidative effect of GLP-1, independent from its effects on blood glucose or body weight.
